# Global proteomics insights for a novel small compound targeting the non-integrin Laminin Receptor in a macrophage cell model

**DOI:** 10.3389/fddsv.2023.1326736

**Published:** 2023-12-17

**Authors:** Abigail Haffner, Manoel Figueiredo Neto, C. Samuel Umbaugh, Tiago J. P. Sobreira, Timothy B. Lescun, Herman O. Sintim, Marxa L. Figueiredo

**Affiliations:** 1 Department of Basic Medical Sciences, College of Veterinary Medicine, Purdue University, West Lafayette, IN, United States,; 2 Bindley Bioscience Center, Purdue University, West Lafayette, IN, United States,; 3 Department of Veterinary Clinical Sciences, College of Veterinary Medicine, Purdue University, West Lafayette, IN, United States,; 4 Department of Chemistry, Institute for Drug Discovery, Purdue University, West Lafayette, IN, United States

**Keywords:** small molecules, PEDF, non-integrin laminin receptor, RPSA, drug discovery, anti-inflammatory, macrophages

## Abstract

**Introduction:**

Monocytes and macrophages are the first barrier of the innate immune system, which interact with agents causing osteoarthritis or other conditions, leading to the release of proinflammatory mediators that exacerbate inflammation.

**Methods:**

The aim of this study was to investigate the proteomic changes in THP-1 monocytes differentiated to macrophages, pre- or -post small compound treatments and in the presence or absence of a proinflammatory stimulus, Lipopolysaccharide (LPS). This study aimed to discover and isolate small compounds that mimic the interaction between Pigment derived growth factor (PEDF) and its 37/67 kDa Laminin receptor (LR) with potential anti-inflammatory activity.

**Results:**

Our results suggested that novel compounds targeting the LR-PEDF interface can be useful for modulating anti-inflammatory effects. Several compounds were selected based on *in silico* docking at the PEDF/LR interface and examined for their ability to reduce IL-1β expression in a macrophage cell model. Compound C3 showed the highest efficacy in reducing IL-1β expression in the presence of LPS proinflammatory stimulus. Proteomics analysis revealed that C3 treatment altered the global proteomic profile of THP-1 activated macrophages, affecting pathways such as MYC targets, oxidative phosphorylation, and mTORC1 signaling.

**Discussion:**

The analysis also highlighted the involvement of key regulators, including RPSA and MYC, and their interactions with other proteins such as ribosome proteins and cell cycle regulators. Furthermore, the downregulated proteome analysis revealed shared and unique pathways affected by the treatments, including processes related to actin cytoskeleton, translation, and the inflammatory response. Protein-protein interaction networks suggested the potential involvement of transcription factors like MYC and the interconnectedness of signaling pathways in mediating such as the effects of the treatments. Overall, these findings provide valuable insights into the potential anti-inflammatory activity and underlying mechanisms of compound C3, emphasizing its relevance for further investigation in the context of inflammatory conditions.

## 1 Introduction

The current state of research in inflammation, particularly in the context of osteoarthritis (OA), is marked by a comprehensive exploration of inflammatory processes and the search for targeted therapeutic interventions. The role of several cells, including monocytes, macrophages, and the inflammatory microenvironment is continuously evolving in our understanding of disease progression. Monocytes and macrophages play a crucial role as the initial defense of the innate immune system, engaging with agents that contribute to conditions such as osteoarthritis and triggering the release of proinflammatory mediators that worsen inflammation. To investigate inflammation, various macrophage cell models are available, among which the THP-1 cell line is notable. THP-1 cells are derived from human monocytic leukemia and can be differentiated into macrophage-like cells by treatment with phorbol 12-myristate 13-acetate (PMA). The resulting cells exhibit many characteristics of mature macrophages, including phagocytosis, cytokine production, and antigen presentation. THP-1 cells are readily accessible and easy to culture, making them a convenient and cost-effective macrophage cell model for studying inflammation in a controlled *in vitro* environment. Novel compounds can be useful for modulating anti-inflammatory effects by regulating the activity of immune cells involved in the inflammatory response, including macrophages. These compounds have the potential to offer targeted and effective therapeutic approaches for treating inflammatory diseases. Recent developments in the field of anti-inflammatory molecules in arthritis, for example, have shown promise for molecules such as kartogenin (krt), which binds core-binding factor β and filamin A to induce expression of SRY box transcription factor 9 (Sox9) to promotes cartilage formation and regeneration (Johnson et al., 2012), and that may be able to reduce levels of pro-inflammatory cytokines (Kwon et al., 2018).

In the present study, we report our recent work with a novel compound, C3, that modulates interactions with the non-integrin Laminin Receptor (also known as RPSA, 37/67 kDa Laminin Receptor, or LR) which has been implicated in joint signaling and the pathogenesis of arthritis. LR is a cell surface receptor that binds to extracellular matrix proteins such as laminin and is involved in a range of cellular processes including adhesion, migration, and signaling. LR is upregulated in synovial tissue and joint fluid from patients with rheumatoid arthritis (RA) and osteoarthritis (OA), suggesting a potential role in the pathogenesis of these conditions (Kane et al., 2019). In addition, LR is involved in the regulation of pro-inflammatory cytokines and chemokines, as well as matrix metalloproteinases. LR targeting may be a promising approach for developing new therapies for inflammatory conditions.

In recent years, various studies have utilized *in vitro* inflammatory models to evaluate the effectiveness of potential therapeutic agents, including sulforaphane, hyperoside, lactate dehydrogenase A, and other promising compounds of various mechanisms, which are still in the early stages of investigation (Davidson et al., 2013; Arra et al., 2020; Sun et al., 2021). Our research team has recently developed a series of novel compounds with potential anti-inflammatory properties. We employed an *in silico* approach, focusing on the interaction between Pigment Epithelium Derived Factor (PEDF) and LR, to initiate this endeavor (Umbaugh et al., 2018). Our rationale was based on PEDF’s relatively large size and low stability at 25°C, which led us to explore the development of smaller, putatively stable molecules as potential therapeutic alternatives. Through our investigations, we identified several small compounds that bind to the docking zone of PEDF at LR, aiming to replicate the beneficial effects of PEDF. PEDF is a 50-kDa glycoprotein known for its anti-inflammatory, anti-angiogenic, anti-oxidative, and anti-tumorigenic activities (Bernard et al., 2009; Wen et al., 2017; Ma et al., 2021). These effects are mediated by PEDF’s interaction with LR (Bernard et al., 2009). Among the identified compounds, “C3” exhibited the highest activity with the lowest effective concentration, effectively mimicking the effects of the interacting region of PEDF (peptide p18) with LR.

The elucidation of PEDF/LR interactions have significantly advanced our understanding of their roles in several diseases; however, knowledge gaps persist in the area of inflammation. For example, studies investigating the role of PEDF and LR in inflammation across various diseases have demonstrated that elevated levels of PEDF can lead to a reduction in pro-inflammatory cytokines, including IL-1β, IL-17A, IL-6, and TNF-α, by inhibiting MAPK p38 and JNK signaling pathways (Matsui et al., 2013; Ma et al., 2021). The precise functions of PEDF/LR interactions in different cell systems remain incompletely understood due to their involvement in complex signaling pathways. However, several reports suggest that these interactions involve MAPK and JNK cascades (Gong et al., 2014; Ma et al., 2021) as well as NF-kB inhibition (Ide et al., 2010). PEDF has been linked to the reduced expression of angiogenic/ECM molecules as well as a network of inflammatory molecules such as TNF, NF-kB, and IL-1 (Zhou et al., 2009; Lu et al., 2014; Matsui et al., 2014). LR also can reduce the expression of several key inflammatory molecules associated with RIPK1 and TNF-mediated apoptosis and inflammatory pathways (Yatim et al., 2015). Also, PEDF and LR, either individually or in combination, have the potential to influence the regulation of multiple inflammatory and ECM-related pathways, with a potential focus on reduced NF-kB signaling. Additionally, the intricate network of inflammatory molecules influenced by PEDF, LR, or their combination expands potential regulatory functions to JUN, SP1, or STAT1/3.

Our group has been focusing on the development of small molecules that could mimic the interaction between PEDF and LR. Specifically, we conducted a thorough analysis of a series of compounds based on the original compound C3. We have predicted that C3 would interact with Histidine-169 of the 37 kDa form of LR (37 LR) through a hydrogen bond, which corresponds to the known binding region for PEDF (Umbaugh et al., 2018). The proposed interaction between C3 and LR is proposed to modulate LR activity and mimic the effects of PEDF. Recent research conducted by our group also has examined the role of C3 in reducing angiogenesis (Umbaugh et al., 2018) and promoting the upregulation of chondrogenic genes in differentiating mesenchymal stromal cell cultures (manuscript in preparation). These initial investigations provide support for the potential therapeutic application of C3 in controlling inflammation and facilitating chondrogenesis. To further explore the effects of C3, we aimed to investigate its impact in conditions mimicking pre-inflammatory and post-inflammatory stimuli using THP-1 cells differentiated into macrophages. For this purpose, we employed global proteomics, a powerful approach that enables a comprehensive characterization of the protein components within biological systems (Cravatt et al., 2007). By employing this technique, we aimed to gain in-depth insights into the protein-level changes and responses induced by C3 in these inflammatory contexts. The current study begins to explore the therapeutic potential of the novel compound C3 in mitigating inflammation in macrophages. Building on insights from the interaction between PEDF and LR, the study extends previous work by identifying C3 as a compound that mimics the anti-inflammatory effects observed in the PEDF-LR interaction. The current study contributes to the evolving landscape of OA research by aiming to address how a novel compound may be utilized in controlling inflammation. This contribution aligns with the broader goal of advancing targeted and more effective therapies for OA, impacting the current state of research in the field.

## 2 Materials and methods

### 2.1 *In silico* screening of compounds

The crystal structure of 37 LR (PDB ID: 3BCH) was cleaned and prepared using AutoDock Vina and AutodockTools, as described (Umbaugh et al., 2018). The Maybridge HitFinder^™^ version 10 library comprising 14,400 drug-like compounds was accessed using “Docking At UTMB” (now “Docking At TACC”), a virtual screening web portal that performs automated docking of precleaned.pdbqt libraries against an input structure (Viswanathan et al., 2014). The *in silico* screening process yielded 24 compounds, selected based on predicted binding scores ranging from −9.3 kcal/mol to −7.9 kcal/mol. This range was chosen to balance strong binding affinity to LR with computational efficiency and diversity in compound selection. Subsequently, these top 24 compounds underwent further evaluation for drug-likeness by a medicinal chemist to yield 5 compounds that we examined in THP-1 cells. This systematic approach ensured a strategic and rational process in identifying compounds with both strong binding affinity and favorable drug-like properties for potential therapeutic applications. The compounds were obtained from Maybridge via Fisher Scientific (now available through Glixx Labs, Hopkinton, MA, United States). Compounds were resuspended in 100% dimethylsulfoxide (DMSO, Sigma Aldrich) to 10 mM and stored at −20°C. Because C3 displayed the most impact on reducing expression of proinflammatory gene IL-1β, we prioritized the remainder of the study using this compound, proceeding to redocking and proteomics characterization. Redocking of C3 was manually performed to the 37 LR crystal structure using AutoDock Vina and AutoDockTools, as described (Umbaugh et al., 2018), revealing a predicted 3.3 Å hydrogen bond with His-169. This interaction site corresponds to the laminin and PEDF binding regions. Modifying His-169 protonation did not affect C3 binding in simulations. In particular, computational redocking indicated C3 engagement at His-169 within the laminin binding region (amino acids 161–180) of 37 LR.

### 2.2 Cell culture

THP-1 human monocytes were purchased from ATCC (Manassas, VA, United States) and cultured in complete media containing 10% fetal bovine serum (FBS, ThermoFisher) with 1 × Dulbecco’s Modified Eagle Medium (DMEM, Corning) and 1 × antibiotic-antimycotic (anti-anti, 100 units/mL penicillin, 100 μg/mL streptomycin, and 0.25 μg/mL amphotericin B; Gibco). The monocytes were differentiated into macrophages with 5 ng/mL phorbol myristate 13-acetate (PMA) for 48 h and pretreated with drugs for 6 h (Park et al., 2007). Lipopolysaccharide (LPS, 10 ng/mL) stimulation was performed for 1 h in complete media and cells were collected for assays.

### 2.3 RNA isolation and qPCR

RNA was isolated from cell pellets using a RNAeasy kit (Qiagen) according to manufacturers’ specifications for tissue samples. 1 μg total RNA were reverse transcribed using the amfiRivert cDNA synthesis master mix (GenDEPOT). Real-time reaction vessels contained 1 μL cDNA template, 2X SYBR Green Master Mix (Applied Biosystems), and 20 μM forward and reverse primers for both experimental and houskeeping gene controls. RT-qPCR was performed on a ViiA7 (Applied Biosystems) instrument, using: 95°C for 3 min; 40 cycles of (95°C for 3 s; 60°C 30 s; 72°C 19 s), and analyzed using Quant Studio Version 1.2 (Applied Biosystems). The target gene assayed was human IL-1β and GAPDH was used as a housekeeping control gene, with primers: IL-1β-f, AAGTACCTGAGCTCGCCAGTGAAA, and IL-1β-r, TTGCTGTAGTGGTGGTCGGAGATT, and GAPDH-f, ACAACTTTGGTATCGTGGAAGG, and GAPDH-r: GCCATC ACGCCACAGTTTC.

### 2.4 THP-1 differentiation and LPS treatment

THP-1 human monocytes were seeded in OptiMEM/1% anti-anti (Gibco) and transfected using Lipofectamine LTX (Invitrogen, Carlsbad, CA) in a 96-well format in white opaque plates. The monocytes were differentiated into macrophages with 5 ng/mL PMA for 48 h and pretreated with drugs for 6 h. LPS (10 ng/mL) stimulation was performed for 1 h and cells were collected for the assay in 40 μL of 1x Reporter Lysis Buffer (Promega, Madison, WI) if used in a luc assay. The samples were freeze-thawed once and shaken for 15 min at room temp, with 5 μL of lysate used in a Beta-gal luminescent assay (Clontech, Mountain View, CA, United States) and the remainder of lysate used for detecting luciferase activity (Luciferase Assay, Promega). The samples were read in a BiotekNeo (Bindley Bioscience Center). For proteomics assays, cells were treated as above, LPS removed at 1 h, cells incubated 24 h, and collected using trypsin:EDTA, pelleted, snap frozen in liquid nitrogen and stored at −80°C until digested for mass spectrometry. A moderate 10 μM C3 dose was chosen from the IL-1β gene expression assays.

### 2.5 Sample preparation

Cells were lysed using the Barocycler NEP2320 (Pressure Biosciences, Inc.) in 50 μL of 100 mM ammonium bicarbonate at 4°C under 35,000 psi for 45 min 100 μg of protein was isolated for digestion using acetone precipitation. After removing acetone, samples were reduced and alkylated, and sequence grade Lys-C/Trypsin (Promega) was used to enzymatically digest the extracted protein. All digestions were carried out in the Barocycler NEP2320 at 50°C under 20,000 psi for 1 h. Peptides were desalted using C18 spin columns (Pierce Biotechnology, Rockford, IL, United States), eluted with 80% acetonitrile (ACN) and 0.1% formic acid (FA), and dried at room temperature in a vacuum concentrator for 1 h. Clean, dry peptides were resuspended in 97% purified water, 3% ACN, and 0.1% FA at a final concentration of 0.2 μg/μL.

### 2.6 Liquid chromatography/tandem mass spectroscopy (LC-MS/MS)

The samples were analyzed using the Dionex UltiMate 3000 RSLC Nano System coupled to the Q Exactive^™^ HF Hybrid Quadrupole-Orbitrap mass spectrometer (ThermoFisher Scientific, Waltham, MA, United States), with a Nano-spray Flex^™^ ion source as described (Umbaugh et al., 2018). Runs involved loading 1 μg total peptide, using a gradient of 0.1% FA in water (solvent A) and 0.1% FA in 80% ACN (solvent B) over 120 min at 35°C. MS data were acquired using a Top20 data-dependent MS/MS scan method with specific settings: injection time of 100 m, resolution of 120,000 at 200 m/z, spray voltage of 2-eV, and an AGC target of 1 × 10^6^ for a full MS spectra scan with a range of 400–1,650 m/z. Precursor ions were fragmented at a normalized collision energy of 27 eV using high-energy C-trap dissociation. MS/MS scans were acquired at a resolution of 15,000 at m/z 200. Dynamic exclusion was set at 30 s to avoid repeated scanning of identical peptides, as described (Opoku-Temeng et al., 2019). Data acquisition was performed monitoring the top 20 precursors at 120,000 resolutions with an injection time of 100 millisecond. Instrument was calibrated at the start of each batch run and then in every 72 h using calibration mix solution (Thermo Fisher Scientific, Waltham, MA). The performance of the spectrometer was evaluated routinely using in-house standards.

### 2.7 Proteomics analyses

Raw files were processed using the MaxQuant (http://maxquant.org/) version 2.1.4.0. The human proteins from UniProt (retrieved on 18 September 2022) and Common contaminants were used as the sequence database. MaxQuant default settings were utilized, except for the following adjustments: “Label-free quantification” (LFQ); “Match between runs” checked; Enzyme Trypsin/P and LysC; Fixed modifications Iodoethanol (C). The MaxQuant result was parsed with an in-house script to remove the common contaminant proteins, extract the LFQ intensity values, and log transform. A missing value was filled when all values of an entire treatment group were missing but all values of the other group were present, the missing values were filled using the lowest intensity value found in the raw data set. A protein was defined to be significantly differentially regulated if it reached a threshold of |log_2_FC|> 0. 3 with *p* < 0.05, based on literature examples where these thresholds could conservatively identify both the changed and unchanged genes/proteins in recent studies correlating proteomic with transcriptomic changes (Mühlhofer et al., 2019).

### 2.8 IDEP9.1, STRING-DB, Enrichr, and DiVenn analyses

IDEP9.1 was utilized to cluster and retrieve the initial signature analyses for the proteomics data (Ge et al., 2018). iDEP is a web-based tool for performing normalization, filtering, clustering, and enrichment analysis based on Gene Ontology (GO), Kyoto Encyclopedia of Genes and Genomes (KEGG), and other Molecular Signatures Database (MSigDB), and can also perform analysis of proteomics data. STRING v9.1 (Szklarczyk et al., 2019; Szklarczyk et al., 2020) was used to map significantly regulated proteins onto protein-protein interaction networks. Gene names corresponding to up- and downregulated proteins were submitted and medium confidence threshold (0.4) used to define protein-protein interactions, as the default from the application, enabling one to determine the reliability of predicted protein-protein interactions. This threshold strikes a balance between sensitivity and specificity, capturing meaningful interactions while minimizing false positives. The selection is often based on empirical testing and the biological context of the study. Gene set enrichment analysis built in STRING with the whole genome background was used to identify enriched gene ontology terms and KEGG pathways. A 0.05 threshold was applied to the p-values after Benjamini-Hochberg correction. Enrichr (Kuleshov et al., 2016; Xie et al., 2021), a gene set enrichment analysis web application, was utilized to compute the enrichment between the input protein lists and the gene sets in the Enrichr library for ranking a term’s relevance to the input list. DiVenn 1.2 (Sun et al., 2019), an interactive and integrated web-based tool was used to visualize and compare the protein lists (*p* < 0.05) from the different triplicate experimental groups, illustrating the number of common and unique protein between groups in a force-directed graph. Clustering analysis using comparison with Enrichr datasets used clustvis (Metsalu and Vilo, 2015). Briefly, rows were centered, clustered using Euclidean distance and average linkage, and unit variance scaling was applied to rows, with values plotted as red representing higher relative expression and blue representing lower relative expression (Rivera-Cruz et al., 2023).

### 2.9 Metascape and ShinyGO enrichment analyses

Gene lists representing the proteins identified were inputted for enrichment analyses in Metascape (Zhou et al., 2019) (accessed 2 Jan 2023). All annotations terms were selected, membership category included all functional sets, pathways, and structural complexes. For enrichment analysis (custom) settings, a minimum overlap of 2 and p of 0.05 were selected for pathways and processes, with the PPI enrichment combined (all) databases. Cytoscape version 3.9.1 was used for the PPI image editing. For ShinyGO, the version 0.76.3 was used (Ge et al., 2020) with the TF.Targets ENCODE.and.ChEA enrichment analysis.

### 2.10 *In vitro* data analysis

Presented as mean ± SD (*n* = 3). 2-tailed *t*-test was utilized with *p*-value ≤0.05 considered statistically significant (*).

### 2.11 Statistics

All assays were run in triplicate with values shown as the mean ± SEM unless otherwise indicated. Student’s t-test was used for pairwise comparisons and one-way ANOVA was used for group comparisons with significance set at *p* < 0.05. In the case of multiple comparisons, the Holm-Sidak test was used, or a false discovery rate was applied.

## 3 Results

### 3.1 *In silico* discovery and isolation of small compounds mimicking the PEDF-LR interaction with potential anti-inflammatory activity

**FIGURE 1:**
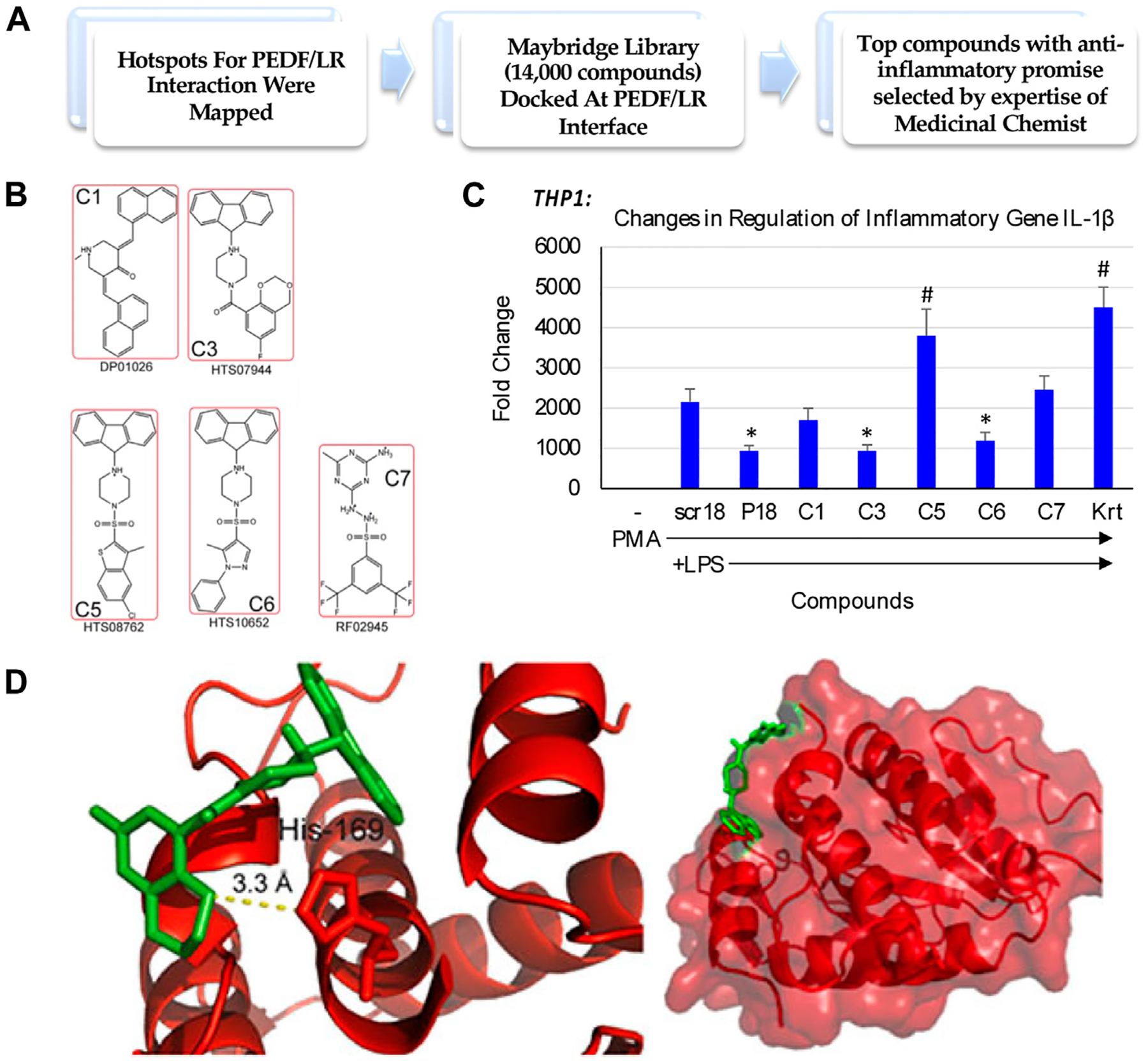
Small molecule discovery for overcoming the challenges underlying endogenous joint repair in Osteoarthritis (OA). **(A)** *In silico* drug design and discovery of 5 compounds selected for potential activity based on other cell culture assays [modified from Umbaugh et al. (2018), under CC BY 4.0]; **(B)** Compound structures; **(C)** Fold change in inflammatory gene expression when THP-1 monocytes are treated with Compounds of Interest. Compound C3 treatment downregulated IL-1β expression even in the presence of strong LPS stimulus relative to a housekeeping gene, GAPDH. PL, PMA-differentiated and treated with LPS. P18, PEDF peptide (Mirochnik et al., 2009). (*, *p* < 0.05 relative to PL); **(D)** Unbiased docking for C3 positions this compound at the interface of the Laminin Receptor at an interaction pocket containing His 169 [modified from Umbaugh et al. (2018), under CC BY 4.0].

We sought to identify a compound that could reduce inflammatory environments, and potentially promote endogenous repair of tissues impacted by inflammatory conditions. With this goal, we set out to test promising compounds that mimicked the interaction of the Pigment derived growth factor (PEDF) and its 37/67 kDa Laminin receptor (LR), identified from a Maybridge library of compounds docked at the PEDF/LR interface (Figure 1A). Some of the top compounds chosen for examining their anti-inflammatory potential are shown in Figure 1B. We examined the activity of these compounds in a macrophage cell model consisting of THP-1 monocytes stimulated with PMA and induced with LPS. One marker examined was IL-1β, and a comparison of the compounds determined their impact on inhibiting IL-1β expression in THP-1 as shown in Figure 1C. In the absence of LPS, the basal levels of IL-1β expression were negligible. A scrambled peptide (scr18) showed the extent of LPS induction of IL-1β relative to baseline. A peptide mimic of PEDF (P18) showed an ability to attenuate IL-1β upregulation by 2-fold (*p* < 0.05). Compared to scr18, the expression of IL-1β was reduced significantly for compounds 3 and 6 (C3 and C6), but not for the other compounds, including Kartogenin (Krt), a small molecule in preclinical translation for osteoarthritis (OA). We selected the compound with the highest ability in reducing IL-1β expression following the LPS proinflammatory stimulus in THP-1 cells differentiated into macrophages to proceed towards global proteomics studies, which was C3. Shown is the C3 compound docked to the LR interaction pocket containing His169 (Figure 1D).

### 3.2 Small compound C3 alters the global proteomic profile of THP-1 activated macrophages

**FIGURE 2:**
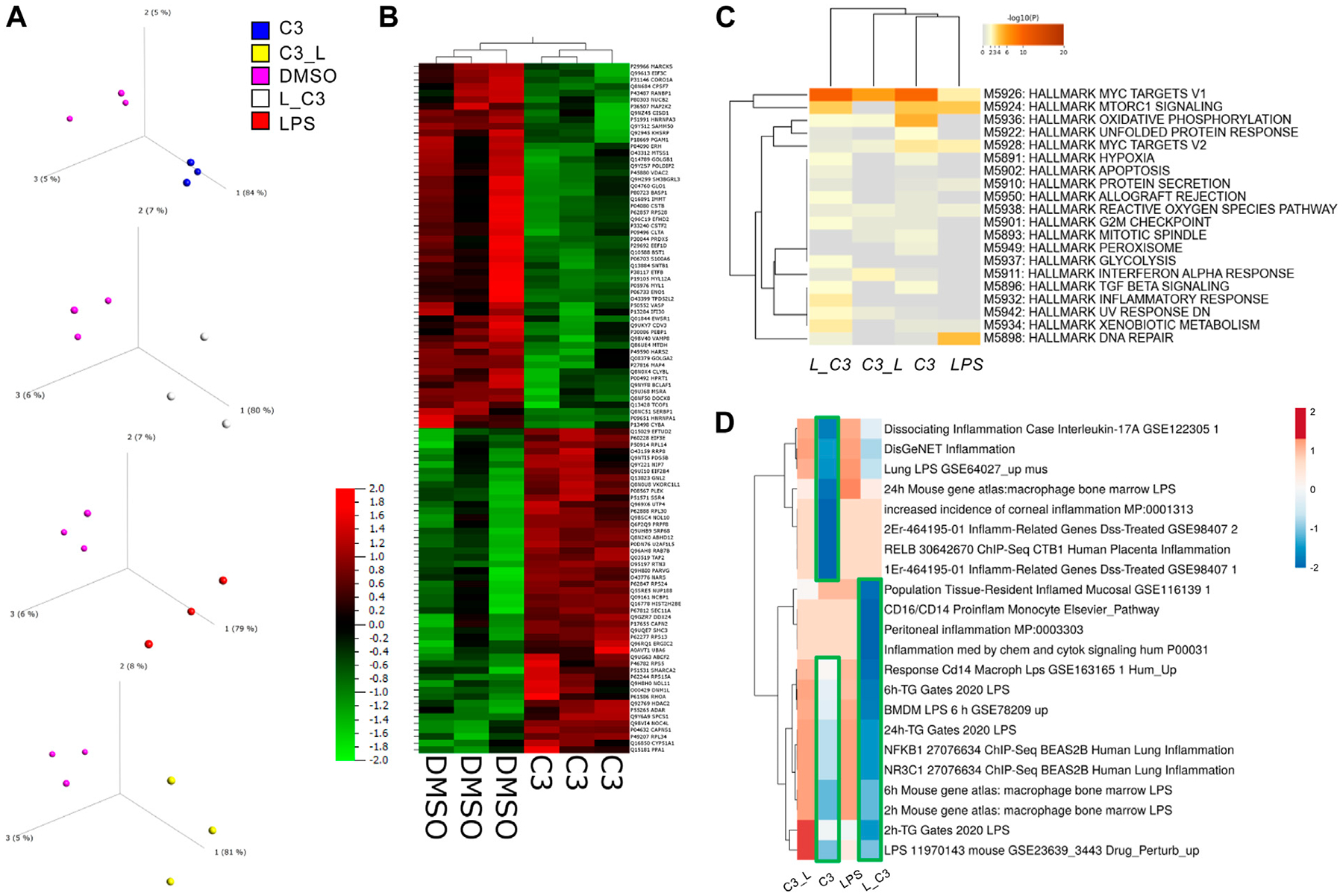
PCA Analysis and global proteomics differences among treated THP-1 macrophages. **(A)** Principal component analysis (PCA) to visualize variance among samples relative to control vehicle (DMSO). **(B)** Clustering analyses showing the global impacts and similarities and differences among samples with C3 and DMSO as examples. **(C)** Enrichment analyses for the combination of significant up and downregulated protein results according to the MSigDB Hallmark gene sets using Metascape. **(D)** Clustering analysis (Clustvis) using comparison with Enrichr datasets relating to inflammation. Green boxes, highlight the downregulated clusters of proteins matching the Enrichr datasets examined. Samples included C3 or LPS alone or with LPS prior to C3 treatment (L_C3) or with LPS following C3 treatment (C3_L).

We examined with principal component analyses each comparison, indicating that each group separated from DMSO signatures (Figure 2A). Clustering analyses indicated a high degree of similarity among the triplicate samples per each treatment group (Figure 2B). We observed a clear separation between the C3 and DMSO treatments. Additionally, some treatments induced a higher degree of upregulated changes (C3) than others, for example, C3 followed by LPS (C3-->LPS) indicating to us that there might be functional differences in pathways regulated by these treatments.

To globally determine the processes or pathways modulated by these treatments, we performed an analysis of key pathways in the MSigDB Hallmark gene set, a collection of 50 refined gene sets, curated from numerous founder gene sets, each representing a specific biological process or state and demonstrating similar patterns of expression (Liberzon et al., 2015). These analyses indicated a key role in the C3-treated groups for MYC targets V1, Oxidative Phosphorylation and to some extent MTORC1 signaling, MYC targets V2, Inflammatory response, Apoptosis, and Hypoxia, among others (Figure 2C).

**FIGURE 3:**
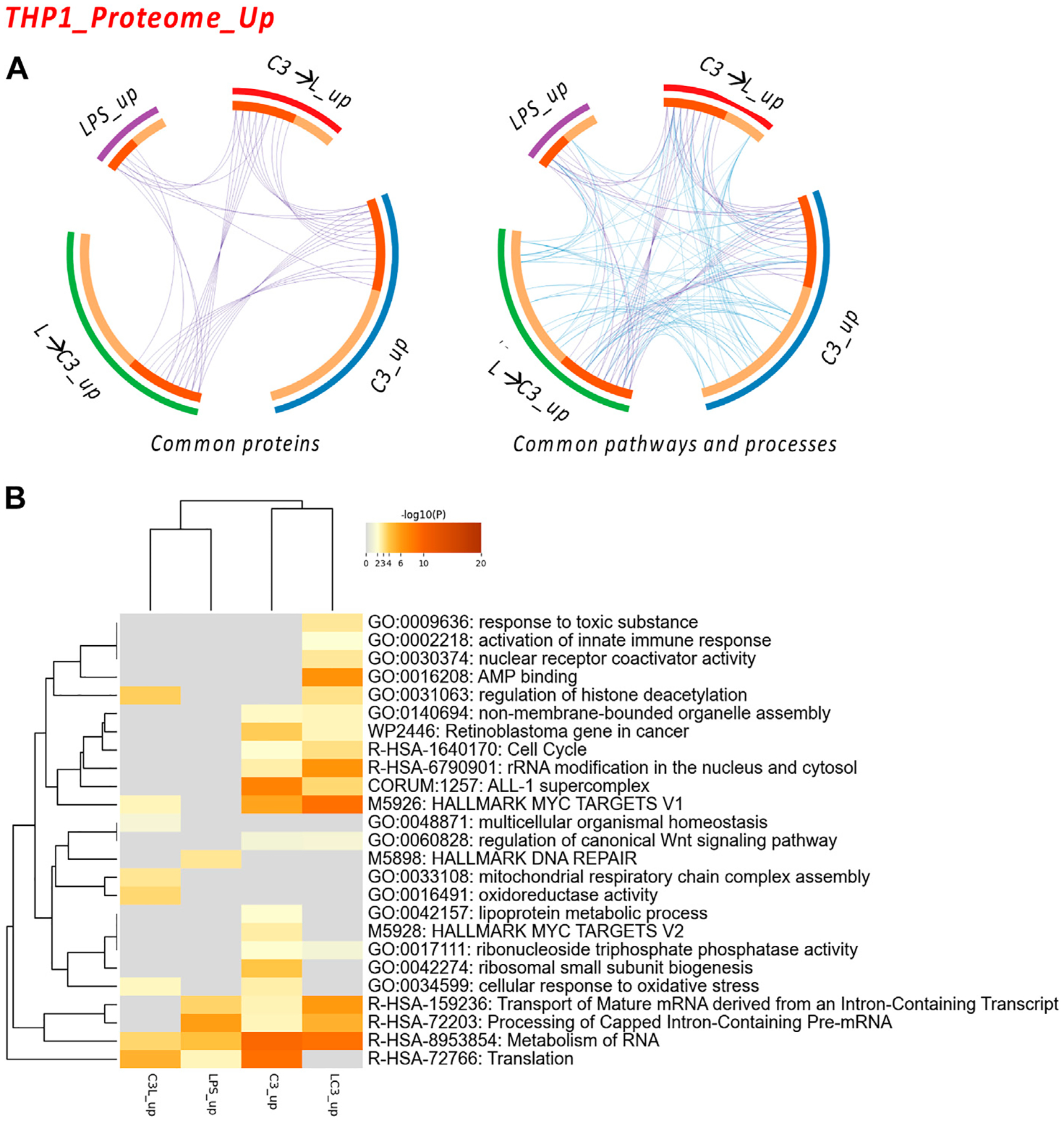
The Proteome Upregulated in THP-1: proteins and processes. Response to LR targeting compound, in the absence (C3) or presence of LPS (C3 followed by LPS, C3→LPS, or LPS prior to C3, LPS→C3) vs. DMSO control. **(A)** Circos plots illustrate the common proteins and pathways and processes among the treated samples. The Circos plot shows how genes from the different input protein lists overlap. On the outside, the arc represents the identity of each protein list. On the inside, the orange color represents the proteins that appear in multiple lists, and the light orange color represents those that are unique to that protein list. Purple lines link the same proteins shared by multiple lists. Blue lines link the proteins that fall into the same ontology term (the term has to be statistically significantly enriched). The greater the number of purple links and the longer the dark orange arcs, the greater is the overlap among the input protein lists. Blue links indicate the amount of functional overlap among the input protein lists. **(B)** Pathway and process enrichment analyses, with −log10 (p) values (color bar) (Metascape). Samples included C3 or LPS alone or with LPS prior to C3 treatment (LC3) or with LPS following C3 treatment (C3L).

Furthermore, an additional analysis was conducted by comparing the datasets with Enrichr, followed by clustering. The results of this analysis indicated that the samples treated with LPS followed by C3, or L→C3, exhibited an inverse correlation with pro-inflammatory datasets, as depicted by the blue cluster in Figure 3D. On the other hand, the samples treated with LPS and C3 followed by LPS (C3→L) both demonstrated a positive correlation with the examined pro-inflammatory datasets, as represented by the orange cluster in Figure 3D.

### 3.3 Pathways relevant to compound activity in different inflammatory conditions in THP-1 cells

We examined the commonalities between the treated groups in terms of proteins, pathways, and processes. The circos plots indicated a high degree of connectivity in terms of pathways and processes (Figure 3A), which was also represented in the heatmap visualization shown in Figure 3B. Among these pathways and processes, the C3 and LPS→C3 samples clustered closely, indicating as common processes Rb gene in cancer, Cell cycle, ALL-1 supercomplex, MYC targets V1, Transport of mature mRNA/Processing and metabolism of RNA, and Translation.

Interestingly, the LPS-treated macrophages clustered closer to the C3→LPS group in this analysis, also showing roles in Translation and RNA metabolism. The C3→LPS group shared pathways with C3, including Regulation of histone deacetylation, MYC targets V1, and Cellular response to oxidative stress, yet was enriched in unique pathways, such as Mitochondrial respiratory chain assembly and Oxidoreductase activity. Finally, the C3 treated macrophages were enriched in the unique pathways of Lipoprotein metabolic process and MYC targets V2.

**FIGURE 4:**
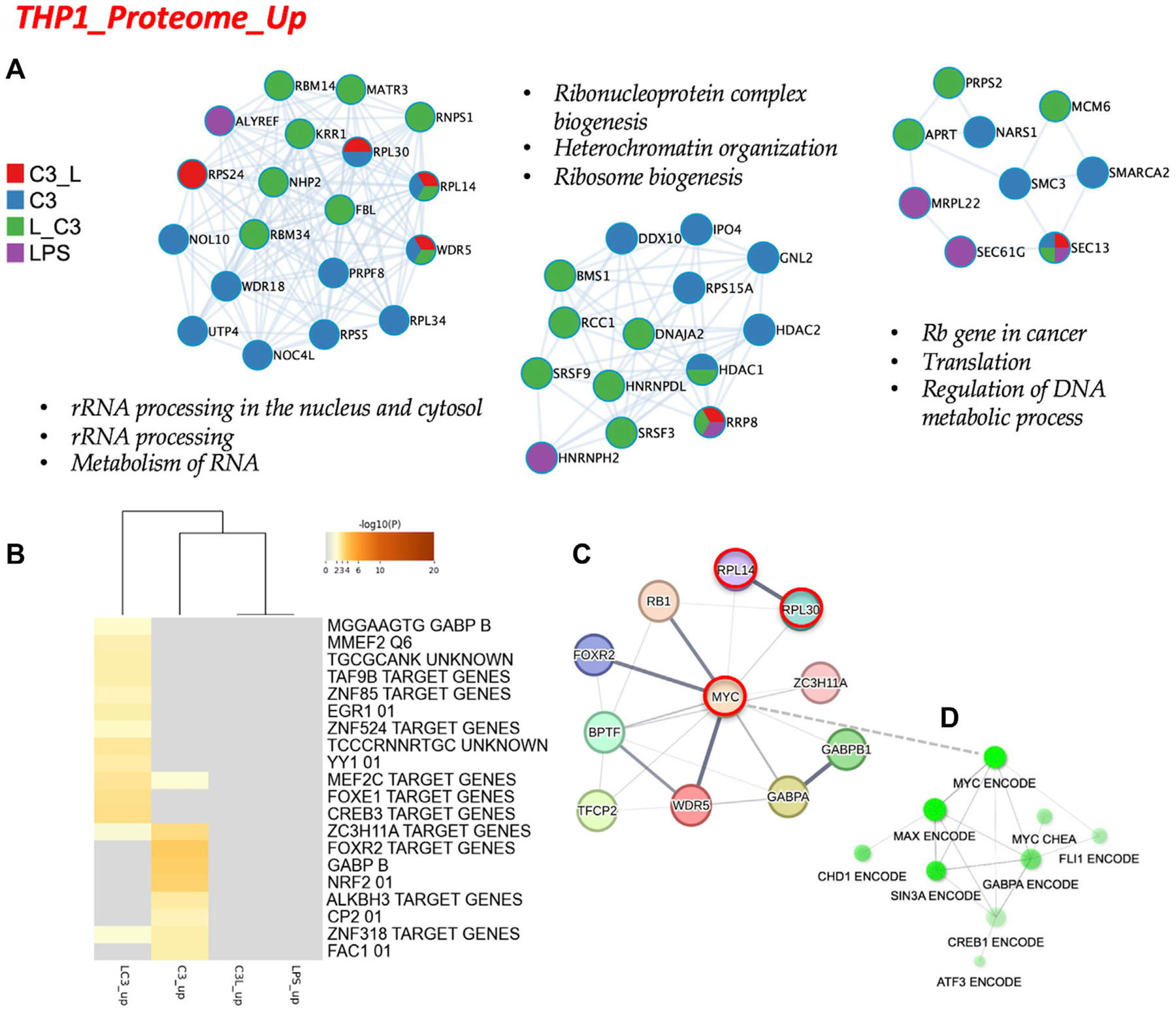
The proteome upregulated in THP-1: protein-protein interaction and regulatory networks. **(A)** The protein-protein interaction enrichment analysis (Metascape). **(B)** Transcription Factor Targets enrichment analyses (Metascape) with −log10 (p) values (color bar). **(C)** Regulatory network for C3 generated in STRING-DB combining information from Figures 4A, B; Red, molecules that also connect to RPSA or PEDF targets. **(D)** Regulatory subnetwork for C3 identified by ShinyGO 0.76.3 using the TF.Target.ENCODE.and.ChEA.Consensus.TFs.from.ChiP-X database. Samples included C3 or LPS alone or with LPS prior to C3 treatment (LC3) or with LPS following C3 treatment (C3L).

Subsequently, we conducted an analysis of the proteome focusing on two categories: the upregulated proteins and the downregulated proteins across the tested conditions. To examine the protein-protein interactions within the upregulated proteome, we employed Cytoscape as a data visualization tool, allowing us to explore similarities and differences among the sample groups. In Figure 4A, we present the obtained networks, with notable changes predominantly observed in the C3 or L→C3 groups. Within these networks, common regulators, such as ribosome proteins (e.g., RPL30 and RPL14), were identified. Furthermore, significant processes related to ribosomal RNA processing and ribosome biogenesis were central to these networks.

Another network highlighted the potential involvement of cell cycle regulation in the regulation of DNA metabolic processes, featuring proteins such as MCM6 and MRPL22. This suggests a potential role of these proteins in coordinating cellular activities. In Figure 4B, the C3 group exhibited closer clustering with the L→C3 group, indicating shared characteristics. Several common transcription factors, including MEF2C, ZC3H11A, and ZNF318, were identified as likely regulators of the protein dataset among these groups. Notably, the C3 group displayed enrichments in several unique regulators, such as FOXR2, GABPB, and NRF2, which may contribute to distinct regulatory mechanisms within this group. These findings provide insights into the protein interactions and regulatory processes associated with the upregulated proteome, highlighting specific regulators and pathways that may be influenced by the treatment with C3 and L→C3.

To explore the broader connections and interactions among the identified regulators from Figures 3, 4, we utilized STRING-DB, and included the RPSA protein, the target of C3. This analysis resulted in the visualization of a network centered around the regulation of MYC, as shown in Figure 4C. Notably, MYC, RPL14, and RPL30 appeared as interactors of RPSA. Furthermore, a subnetwork analysis using data from CHEA and ENCODE revealed connectivity within a subnetwork involving MYC, MAX, SIN3A, and FLI1, specifically for the set of upregulated proteins. This is depicted in Figure 4D. These findings suggest the involvement of these regulators in coordinating the cellular processes indicated in Figure 4 and potentially influencing the observed upregulation of proteins in response to the treatments. Overall, these results provide insights into the interconnectedness of key regulators and highlight the potential involvement of MYC and its associated interactors, i.e., the MYC/MAX/SIN3A/FLI1 subnetwork, in mediating the effects of the tested conditions on protein expression.

**FIGURE 5:**
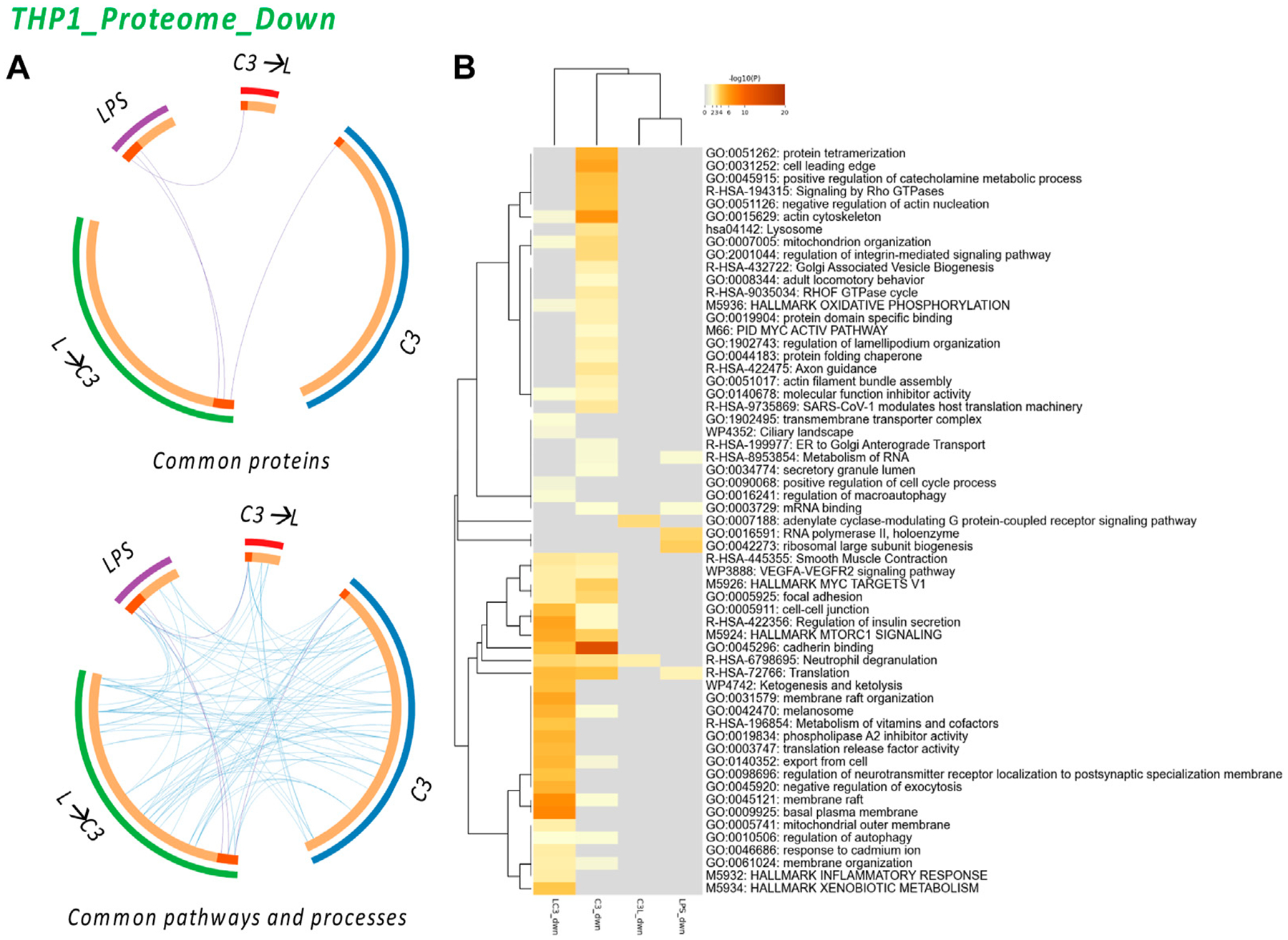
The Proteome Downregulated in THP-1: proteins and processes. Response to LR targeting compound in the absence (C3) or presence of LPS (L), either after C3 treatment (C3→L) or prior to C3 treatment (L→C3) vs. DMSO control. **(A)** Circos plots illustrate the common proteins and pathways and processes among the treated samples. **(B)** Pathway and process enrichment analyses, with −log10 (p) values (color bar) (Metascape). Samples included C3 or LPS alone or with LPS prior to C3 treatment (LC3) or with LPS following C3 treatment (C3L).

In contrast, to explore the shared characteristics among the treated groups regarding the downregulated proteins, we used circos plots to visualize the connectivity of pathways and processes, as depicted in Figure 5A. Consistent with this, the heatmap visualization in Figure 5B also demonstrated a high degree of connectivity among the downregulated pathways and processes across the various treatment groups. Notably, the C3 and LPS→C3 samples displayed the highest number of commonly downregulated processes. This suggests a potential convergence in the regulatory mechanisms and pathways affected by C3, even in the presence of LPS.

The analysis revealed several common processes shared among the treated groups, including Actin cytoskeleton, Mitochondrion organization, MYC V1 targets, VEGF-VEGFR2 signaling pathway, Cell-cell junction, MTORC1 signaling, Cadherin binding, Translation, Membrane raft, Autophagy, and Membrane organization. These processes were found to be downregulated across multiple groups, indicating their potential involvement in the response to C3. Additionally, the C3 sample exhibited unique pathways, such as Protein tetramerization, Cell leading edge, Signaling by Rho GTPase, Negative regulation of actin nucleation, Lysosome, Integrin pathway, and Protein folding. These pathways may represent specific regulatory mechanisms influenced by the C3 treatment. Interestingly, the analysis indicated that only the LPS→C3 group downregulated processes associated with the Inflammatory response and Xenobiotic metabolism/response to cadmium, Ketogenesis, Metabolism of vitamins and cofactors, and other processes related to the plasma membrane. The C3→L group showed downregulation in only two enriched processes: Adenylate cyclase-modulating G protein-coupled receptor signaling and Neutrophil degranulation (common to all C3 groups). In comparison, the LPS-only group shared Translation processes with two C3 groups and Metabolism of RNA with at least one C3 group. It also exhibited unique processes related to RNA polymerase II and Ribosomal large subunit biogenesis. These findings highlight both shared and distinct pathways affected by the treatments, providing insights into the potential mechanisms and processes involved in the protein expression changes detected.

**FIGURE 6:**
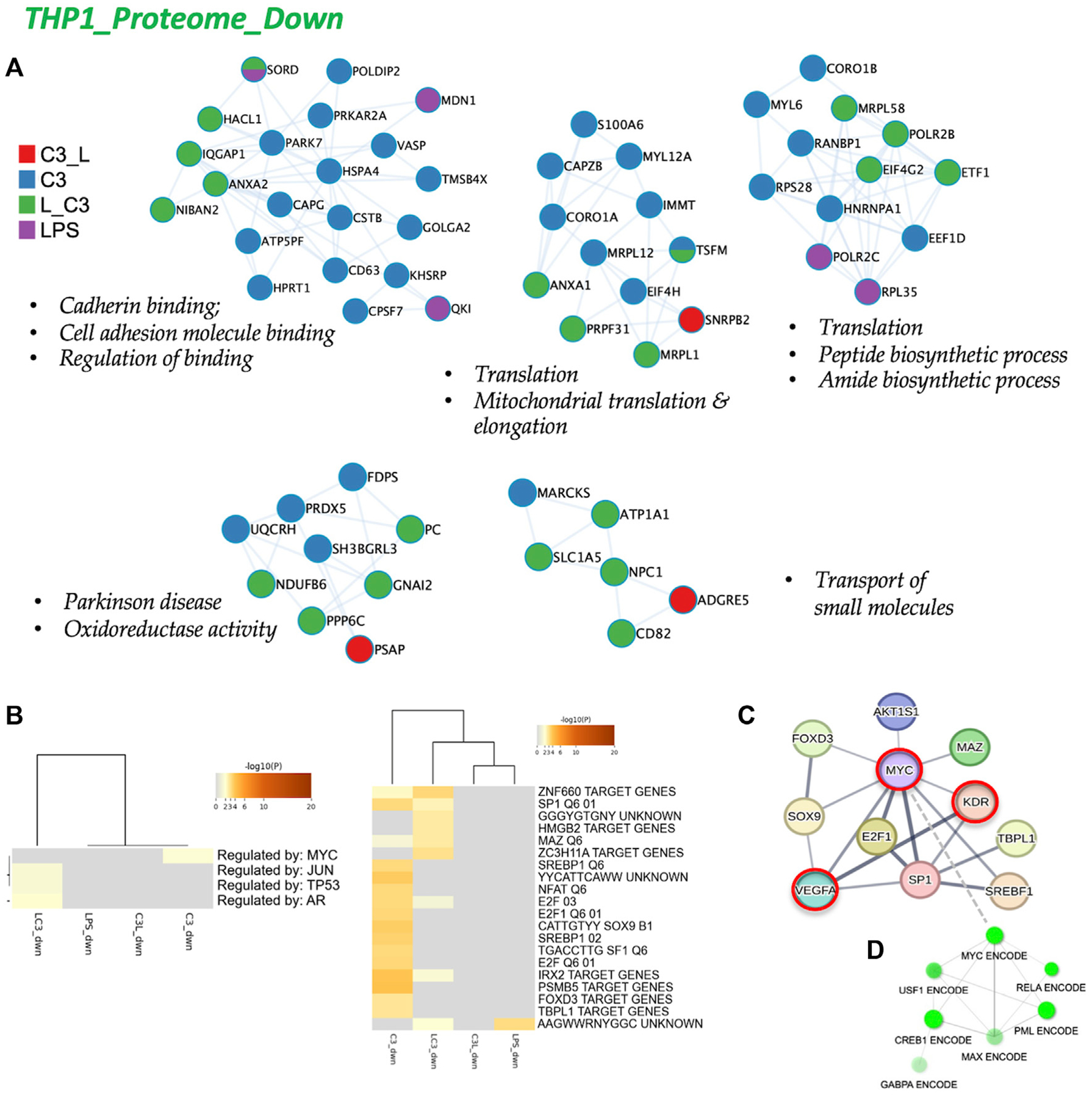
The proteome downregulated in THP-1: protein-protein interaction and regulatory networks. **(A)** The protein-protein interaction enrichment analysis (metascape.org). **(B)** Transcription Factor Targets enrichment analyses (Metascape) with −log10 (p) values (color bar). **(C)** Left panel, Regulatory network for C3 generated in STRING-DB combining information from Figures 5, 6; Red, molecules that also connect to RPSA or PEDF targets. Right panel, regulatory subnetwork for C3 identified by ShinyGO 0.76.3 using the TF.Target.ENCODE.and.ChEA.Consensus.TFs.from.ChiP-X database. Samples included C3 or LPS alone or with LPS prior to C3 treatment (LC3) or with LPS following C3 treatment (C3L).

To analyze the protein-protein interactions involved in the downregulated proteome, we utilized Cytoscape as a visualization tool to explore similarities and differences across the sample groups. In Figure 6A, we present the networks highlighting prominent changes observed in the C3 or L→C3 groups. Notably, significant downregulated processes were centered around Cell adhesion binding, Translation, Mitochondrial translation and elongation, Peptide and amide biosynthetic processes, Oxidoreductase activity, and Small molecule transport. Figure 6B summarizes the Metascape analysis, focusing on transcription factor enrichment. In the left panel, MYC was identified as a likely regulator of proteins in the C3 group, while Jun, p53, and AR were associated with proteins in the L→C3 group according to the TTRUST analysis. In the Transcription factor targets analysis (right panel, Figure 6B), the C3 group clustered closely with L→C3, sharing several predicted regulators such as ZNF660, SP1, MAZ, E2F, and IRX2. The C3 group exhibited additional unique regulators, including SREBP1, NFAT, E2F1, SOX9, SF1, PSMB5, FOXD3, and TBPL1.

To gain a broader perspective on the interconnectedness of these regulators, we inputted them into STRING-DB, resulting in a network centered on MYC regulation (top panel, Figure 6C). Interestingly, MYC was identified as an interactor of LR or RPSA, the target of C3, and KDR (VEGFR2) and VEGFA were identified as interactors of PEDF. Furthermore, using CHEA and ENCODE data, a subnetwork involving MYC, RELA, CREB, and PML was revealed for the set of downregulated proteins (Figure 6D). These findings provide insights into the regulatory mechanisms and potential interactions underlying the observed downregulated protein processes, highlighting the involvement of key transcription factors such as MYC and the interconnectedness of various signaling pathways.

### 3.4 Mapping the interactivity with MYC targets

**FIGURE 7:**
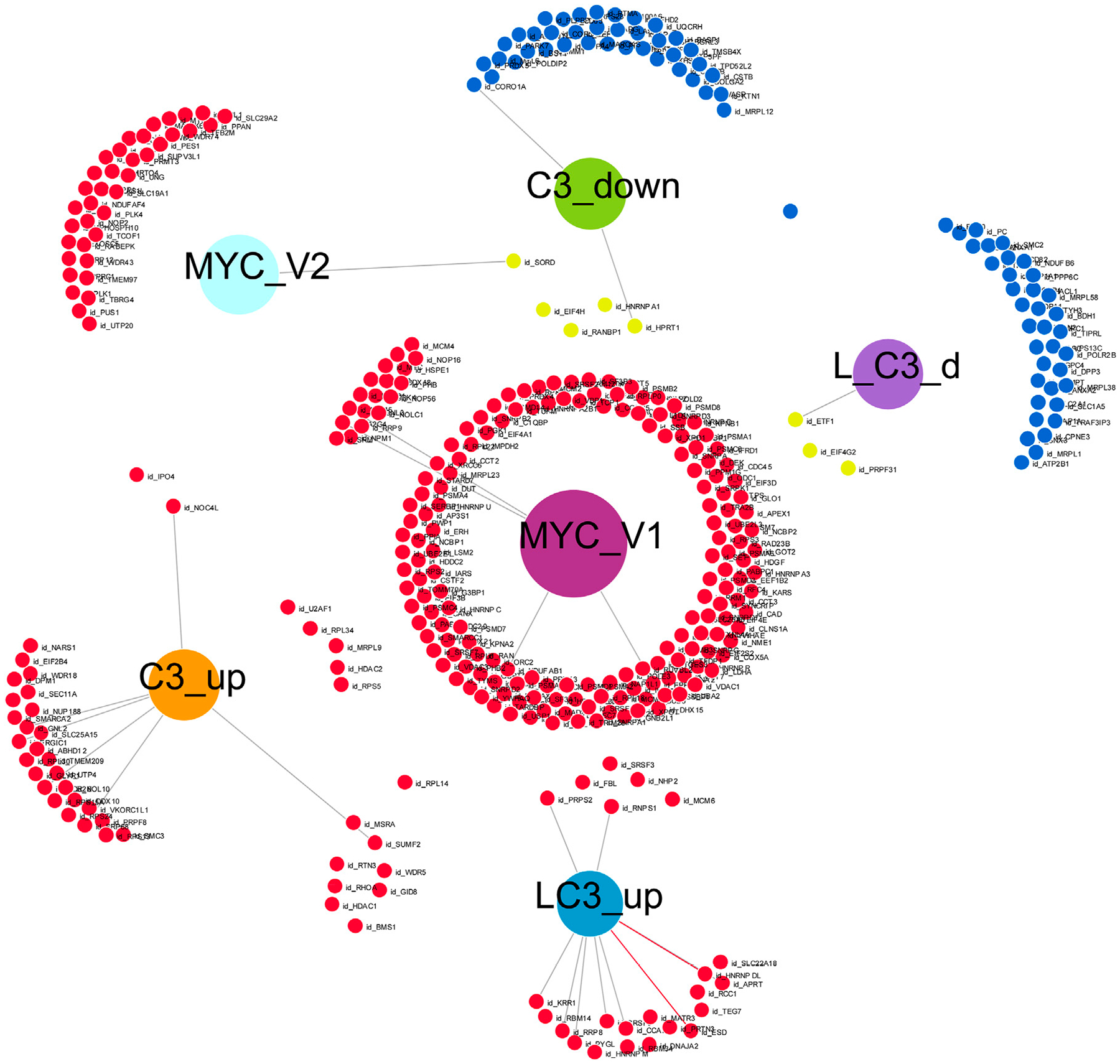
Visual representation of the connectivity of key C3 groups in the absence or presence of LPS (L), with Myc target genes, generated with Divenn (Sun et al., 2019). Samples included C3 alone or with LPS prior to C3 treatment (L_C3).

In our final analysis, summarized in Figure 7, we investigated the connectivity among four groups treated with C3 (C3_up, C3_down, L→C3_up, and L→C3_down), which exhibited the highest number of protein regulation changes, particularly in relation to the MYC V1 or V2 targets from the Hallmark datasets. To conduct this analysis, we utilized DiVenn (Sun et al., 2019), which enabled the following observations. Among the proteins downregulated by C3, four (EIF4H, RANBP1, HNRNPA1, and HPRT1) overlapped with MYC V1 targets. Additionally, three proteins downregulated by L→C3 (ETF1, EIF4G2, and PRPF31) exhibited overlap with MYC V1 targets, and one protein (SORD) downregulated by L→C3 overlapped with MYC V2 targets. On the other hand, five proteins upregulated by C3 (U2AF1, RPL34, MRPL9, HDAC2, and RPS5) were found to overlap with MYC V1 targets, while two proteins (IPO4 and NOC4L) overlapped with the MYC V2 dataset. Furthermore, one protein (RPL14) was common between the upregulated samples of both C3 and L→C3 groups. Notably, six proteins were shared between the upregulated proteins of L→C3 group and MYC V1 targets. Overall, this analysis provides a more detailed overview of the interactivity among the key treatments and their differential interaction with MYC targets. It highlights specific proteins that are regulated by C3 and L→C3 treatments and their connection to MYC, offering valuable insights into the molecular interactions involved in the observed protein regulation changes.

Further analysis using Enrichr revealed specific processes regulated by the key proteins that were downregulated by C3, with mapping to various pathways and processes. For example, these proteins were found to be associated with Purine salvage/metabolism and Azathioprine (Reactome), EGFR1 pathway and Cycling of Ran in nucleoplasmic transport (Bioplanet), as well as Purine metabolism and Nucleotide metabolism (WikiPathway). These processes are likely regulated by MYC and EIF1B, MCC, or IKBKE as indicated by the protein-protein interaction (PPI) hub proteins. On the other hand, the downregulated proteins in the L→C3 group were associated with processes such as Metabolism of RNA and Oncogene-induced senescence (Reactome), Internal ribosome entry pathway and Eukaryotic protein translation (Bioplanet). These processes may be regulated through MYC V1 targets and CDK2, GSTK1, and PTP4A3 (PPI hub proteins). In terms of upregulated proteins related to MYC V1 and C3, they were found to be associated with Translation (Reactome) and Cytoplasmic ribosomal proteins (Bioplanet), potentially regulated through MYC V1 and GABARAPL2, GABARAP, or CSNK2A1 (PPI hub proteins). The two proteins upregulated by C3 that were related to the MYC V2 dataset were mapped to processes such as rRNA modification in the nucleus and cytosol (Reactome), with potential PPI hub protein regulators GSTK1 and MED19. Lastly, the protein commonly upregulated by C3 and L→C3, RPL14, was found to map to processes such as Peptide chain elongation (Reactome) and Ribosome (KEGG), possibly indicating its involvement in protein-protein interactions with ASF1, MAP3K14, or EIF2C1 as part of a PPI hub. These findings provide insights into the specific pathways and processes that are regulated by the identified proteins and their potential interactions with key regulators such as MYC and other PPI hub proteins.

## 4 Discussion

In this study, our group investigated the effect of a novel small compound, C3, targeting the Laminin Receptor (LR or RPSA), on human monocytes differentiated into macrophages and stimulated with LPS to induce inflammation. We compared the performance of C3 with other compounds and found that C3 showed promise in reducing the expression of IL-1β, a major inflammatory mediator. Interestingly, kartogenin (krt), under preclinical development for osteoarthritis, was not able to reduce IL-1β gene expression induced by LPS in the macrophages in our study. The literature suggests krt still is very promising, since it inhibits IL-1β expression in chondrocytes (Ye et al., 2023).

To further understand the mechanisms underlying C3’s anti-inflammatory activity, we conducted global proteomic analysis. We observed significant changes in several pathways, including MYC targets, oxidative phosphorylation, inflammatory response, apoptosis, and hypoxia, among others. These findings suggested that C3 treatment promotes the upregulation of ribosome proteins and processes related to ribosomal RNA processing and ribosome biogenesis. MYC, RPL14, and RPL30 were identified as interactors of RPSA, the target receptor of C3, in STRING-DB/Biogrid. This study also revealed the interplay between MYC and other transcription factors such as SIN3A, GABPA, FLI1, and CREB1. These factors are known for their roles in cell proliferation, differentiation, and survival but may also have implications in modulating inflammation and immune responses. MYC, in complex with MAX, can either activate or repress target genes depending on the cellular context. Additionally, SIN3A can recruit histone deacetylases to repress gene expression, while RB1 can inhibit cell cycle progression by binding to E2F transcription factors. GABPA, by interacting with the MYC-MAX complex, enhances its transcriptional activity, promoting cell proliferation and survival. The analyses suggest that C3 has the potential to regulate inflammatory pathways through its interactions with MYC and related factors. Further research is required to fully elucidate the specific roles of these factors and their involvement in different inflammatory conditions. C3 holds promise as an anti-inflammatory therapy and may contribute to the development of novel treatments for various inflammatory and immune-related diseases. More recent work links ribosome biogenesis modulation with restriction of innate immune responses and reductions in pro-inflammatory cytokines, unexpectedly regulating inflammation (Bianco and Mohr, 2019). Interestingly, anti-ribosome biogenesis therapeutics also can help reduce the inflammatory milieu in mice with induced endometriosis (Chang et al., 2022).

The interplay between MYC V1 and V2 splice variants, along with their binding partners, suggests a potential role for MYC in modulating inflammation. The MYC-MAX complex can activate or repress target genes depending on partner proteins such as SIN3A, GABPA, FLI1, and CREB1, which may impact immune responses and inflammatory processes. The specific effects on inflammation and IL-1β expression can be complex and context-dependent, influenced by the balance of activating and repressive mechanisms regulated by these proteins. For example, ribosomal proteins RPL14 and RPL30, regulated by MYC, are involved in ribosome assembly, and play a role in cell growth and proliferation. MYC can impact inflammation positively or negatively through ribosome biogenesis, depending on the cellular context.

The downregulation of mediators like RELA/CREB, possibly through interaction with PML, may have an anti-inflammatory effect by inhibiting NF-kB and CREB signaling pathways. PML can sequester RELA/p65 in the cytoplasm, preventing its nuclear translocation and subsequent activation of pro-inflammatory genes.

The sequencing of inflammatory stimuli, such as LPS and pretreatment with C3, for example, could influence the activation of V1 and V2 targets. It is known that MYC V1 can directly bind to the promoter region of IL-1β and upregulate its expression, while MYC V2 may play a role in the regulation of inflammation, but the specific targets remain uncharacterized. Both MYC V1 and V2 have been associated with pro-inflammatory processes, yet there is evidence to suggest that they may also be involved in anti-inflammatory processes, by regulating the expression of genes involved in both pro- and anti-inflammatory responses. In conclusion, MYC, in conjunction with its splice variants and binding partners, appears to play a role in modulating inflammation and immune responses. The specific effects on inflammation and target gene regulation vary depending on the splice variant, cellular context, and interacting partner proteins. Ribosomal proteins, mediators, and hub proteins are also implicated in the regulation of inflammatory processes. Further research is needed to fully elucidate the mechanisms and therapeutic potential of targeting MYC and its associated proteins in inflammation-related diseases.

Further analysis identified specific processes and pathways influenced by treatment with C3. Upregulated proteins were linked to Translation and Cytoplasmic ribosomal proteins. Hub proteins including GSTK1, MED19, ASF1, MAP3K14, EIF2C1, CDK2, GSTK1, PTP4A3, and IKBKE were identified. In particular, these proteins that overlap with MYC targets might hold functional significance in inflammation and tissue repair. For instance, GSTK1 contributes to cellular detoxification, whereas MED19 is involved in transcriptional regulation. ASF1 plays a role in chromatin dynamics, and MAP3K14 is a key player in the MAPK signaling pathway. EIF2C1 is crucial for post-transcriptional gene regulation, and CDK2 is a key cell cycle regulator. PTP4A3 modulates cell growth and migration, and IKBKE activates the NF-κB signaling pathway. Together, these proteins suggest a diverse set of cellular functions that potentially coordinate responses during inflammation and tissue repair, making them promising targets for further investigation. Interestingly, the expression changes and processes influenced by C3 treatment also were linked to Azathioprine, a medication used for rheumatoid arthritis to promote immunosuppression, suggesting an anti-inflammatory role for C3. Pathways influenced by C3 that might be associated with tissue repair may include the downregulation of proteins associated with MYC V1 targets, suggesting potential control over excessive cell proliferation. The impact of C3 on purine salvage/metabolism and EGFR1 pathway proteins might suggest it could contribute to DNA repair, regulating cell signaling during tissue repair. Disruption of nucleoplasmic transport could affect gene expression and repair coordination also. Upregulation of translation-related proteins might indicate enhanced protein synthesis, which is essential for cellular repair. Collectively, these findings suggest that C3 has the potential to play a multifaceted and beneficial role in tissue repair processes by orchestrating specific cellular responses.

Ongoing and future studies by our group are focusing on investigating whether C3, in addition to its anti-inflammatory properties, influences MSC chondrogenic differentiation in human and equine cells. We continue to explore the effects of C3 on MSCs in terms of their differentiation potential, extracellular matrix synthesis, and cartilage-related gene expression. Future applications of C3 are likely to require specialized formulations that can be targeted to specific tissues or designed for prolonged presence within joints, as shown for krt, that can be targeted or designed for persistence within joints. Recent advances have included nanocarriers that can home to cartilage or inflamed joints, for example (He et al., 2022). Our future objectives involve developing a drug delivery system for C3 in OA treatment and optimizing C3 through structure-based drug design for effective compound development for equine and human applications.

In summary, our study suggests that the small compound C3, which targets the non-integrin Laminin Receptor, shows promise as an anti-inflammatory therapy. The findings suggest that C3 has the potential to modulate inflammatory pathways through its interactions with MYC and related factors. Further research is required to fully elucidate the specific roles of these factors and their involvement in different inflammatory conditions. C3 holds promise as an anti-inflammatory therapy, and perhaps it may serve as a disease-modifying antirheumatic drug (DMARD), contributing to the current landscape of novel treatments for various inflammatory and immune-related diseases.

## Data Availability

The datasets presented in this study can be found in online repositories. The names of the repository/repositories and accession number(s) can be found below: https://massive.ucsd.edu/ProteoSAFe/dataset.jsp?accession=MSV000092882.
